# Protective role of *Lactobacillus* *plantarum A7 *against irinotecan-induced genotoxicity

**Published:** 2016

**Authors:** Soheila Sepahi, Abbas Jafarian-Dehkordi, Maryam Mirlohi, Kobra Shirani, Mahmoud Etebari

**Affiliations:** 1 *Department of Pharmacology, Isfahan Pharmaceutical Sciences Research Center, School of Pharmacy and Pharmaceutical Sciences, Isfahan University of Medical Sciences, Isfahan, Iran*; 2 *Food Security Research Centre, School of Nutrition and Food Sciences, Isfahan University of Medical Sciences, Isfahan, Iran*; 3 *Department of Pharmacodynamy and Toxicology, Faculty of Pharmacy, Mashhad University of Medical Sciences, Mashhad, Iran*

**Keywords:** *Irinotecan*, *Lactobacillus plantarum A7*, *Anti-genotoxicity*, *HepG2*, *Comet assay*

## Abstract

**Objective:**

Irinotecan is a botanical derivative and an anti-cancer drug with cytotoxic and genotoxic effects. The present study evaluated the effect of *Lactobacillus plantarum A7* on the genotoxic activity of irinotecan in a hepatocellular carcinoma cell line (HepG2) by comet assay.

**Materials and Methods:**

HepG2 were incubated with irinotecan (100 µM), heat-killed cells (0.025 µg/ml) + irinotecan (100 µM), and cell-free supernatants (0.5 and 1 µg/ml) of *L. plantarum A7* + irinotecan (100 µM). Phosphate buffered saline (PBS) was used as negative control.

**Results:**

Irinotecan was shown to induce DNA damage in HepG2 cells. The results showed that heat-killed cells (0.025 µg/ml) and cell-free supernatants (0.5 and 1 µg/ml) of *L. plantarum *significantly reduce irinotecan- induced DNA damage.

**Conclusion:**

Our results indicate that *L. plantarum A7 *can decrease the genotoxic effects of irinotecan in HepG2 cells,* in vitro*. This finding may be supportive for the optimization of therapeutic efficacy in irinotecan treatment.

## Introduction

Irinotecan (CPT-11), derived from the Chinese shrub *Camptotheca acuminata*, is a globally approved agent for the treatment of patients with metastatic colorectal and ovarian carcinoma (Cragg and Newman, 2005). It causes genotoxicity through inhibition of DNA replication by acting upon DNA topoisomerase I enzyme (Santos et al., 2000). In spite of excellent therapeutic effect of irinotecan toward cancer tissue, the DNA of non-cancer cells is also subjected to damage during chemotherapy which can lead to myelosuppression, hepatotoxicity and diarrhea (Lévesque et al., 2013).

During recent years, there has been considerable interest in dietary agents that can influence the response to chemotherapy as well as the development of adverse side effects that are resulted from treatment with antineoplastic agents. In this regard, prebiotics have received particular attention (Caldini et al., 2005, Kontek et al., 2010). Probiotics are defined as live microbial feed supplements that improve health of host when administered in adequate number. Strains of lactobacilli occur in large numbers in various fermented products such as yoghurt and cheese. Lactobacilli are considered to have gastrointestinal protection, serum cholesterol reduction, immunostimulant, anti-mutagenicity, and anti-genotoxicity activities (Fazeli et al., 2010; Parvez et al., 2006).

The ability of probiotics to decrease the genotoxic activity of chemical compounds such as mutagenic heterocyclic amines, Aflatoxin B1, and pyrolyzates have been documented in previous studies (Apás et al., 2014). The protective mechanisms of probiotics are reduction of bacterial enzyme activities involved in carcinogen formation, direct inhibition of tumorigenesis by their metabolites, mutagen binding on cell components, and mutagen bioconversion binding on cell components (Kahouli et al., 2013; Raman et al., 2013). 

Anti-genotoxicity activities of lactic acid bacteria may vary from strain to strain and there is a need to find new probiotic strains with genoprotective effects in *in vitro* studies (Kumar et al., 2015). *L. plantarum A7* is one of these strains that has been isolated from fecal flora of healthy infants (Sadeghi-Aliabadi et al., 2014). This study aimed to evaluate anti- genotoxicity activities of *L. plantarum A7* against irinotecan-induced DNA damage in HepG2. To examine whether cellular fractions or produced metabolites by the tested strains could inhibit genotoxic activity; both heat-killed (HK) cells and cell-free supernatants of *L. plantarum A7* were investigated in two independent series of experiments.

## Materials and Methods

**Bacterial culture**

*Bacteria strain and culture medium*

*L.* *plantarum A7 *strain was provided from the microbial collection of food microbiology laboratory of Industrial University of Isfahan*. *Overnight cultures were prepared using 1% inoculums of each strain into deMan-Rogosa-Sharp (MRS) broth (Merck, Germany) containing 0.05% L-Cysteine Hydrochloride Monohydrate (DAE JUNG, Korea) and incubated for 18 hr in anaerobic conditions at 37 °C. To prepare the active cultures, they were sub-cultured at least three times before the experiment (Sadeghi-Aliabadi et al., 2014).

**Preparation of freeze-dried cell-free supernatant**

Supernatant was obtained by centrifuging (Hettich, Germany) the medium at 10000 rpm for 15 min at 20˚C. Centrifuged supernatant was filtrated through a 0.22 µm micro filter. Cell free supernatants were subsequently subjected to lyophilisation (Christ, alpha 2-4 LD plus, Germany). Solutions of freeze-dried supernatant in sterilized PBS were prepared at different concentration to be tested by comet assay (Sadeghi-Aliabadi et al., 2014).

**Preparation of heat-killed cells**

Bacterial pellets, resulted from centrifugation, were used for the preparation of heat-killed cells as follows: precipitates were washed twice with PBS and incubated for 1 hr at 95°C. To confirm the killing of all bacteria, one sample was cultured in MRS broth (Sadeghi-Aliabadi et al., 2014). The killed bacteria were freeze-dried and kept in airtight packaging at 25°C. The lyophilized killed cells were re-suspended in PBS to prepare the solutions of different concentrations. Solutions with the optical densities of 0.025, 0.05 and 0.1 at 620 nm were used in comet assay. 

**Genotoxin**

Irinotecan, HCl-trihydrate (20 mg/mL) (Campto®) was purchased from actavis Co (Romania). To prepare the stock solution of irinotecan, it was diluted using sterile PBS.

**HepG2 cell culture**

HepG2 cells were cultured with RPMI 1640 medium (Biosera, France) which was supplemented with 5% foetal bovine serum (Biosera, France) and 1% penicillin/streptomycin (Biosera, France) and maintained in a humanized atmosphere (90%) containing 5% CO2 at 37 °C.

To find the least genotoxic dose, the cells were exposed to different concentrations of irinotecan for 1 hour. Then, to explore the genotoxic concentrations of cell-free supernatant and heat-killed cells of *L. plantarum A7*, the cell were incubated with bacteria for 1 hr. 

To study protective effects of probiotics, cells were exposed to safe concentrations of cell-free supernatant and heat-killed cells of the introduced bacteria. Having rinsed the bacteria, they were exposed to lowest genotoxic concentration of irinotecan. As described in the previous studies, the plates containing medium were washed with sterile PBS. To detach the remaining treated cells, trypsinization was performed (trypsin EDTA, Biosera, France). Transferring the medium to a 15 ml falcon tube, centrifuging (at 1800 rpm for 5 min) were the two measures taken respectively for the removal of Trypsin. Finally, 1 ml of the above-mentioned medium was added to each falcon tube to be able to take the next stages of the comet assay. For all experiments, negative control (PBS) was included (Jafarian et al., 2014).

Trypan blue dye exclusion method was used to determine cell viability. At least, 90% of cell viability was obligatory to implement the comet assay.

**Comet assay**

Comet assay was used in alkaline conditions (pH>13) according to the technique proposed by Singh et al., (1998). For this purpose, at least 10^6 ^cells that were previously prepared in cell culture phase were used. Then, 300 μl of cells suspension was mixed with 1 ml Low Melting Point (1%LMP) agarose (Sigma, USA). 

The resulted mixture was layered onto slide, pre-coated with NMA (Normal Melting agarose) (Cinnagen, Iran); cover slips covered them. Having been maintained at 4°C for 10 min for solidification, slides coverslip was removed. Then, slides were submerged in lysis solution (2.5 M NaCl,100 mM EDTA, 0.2 M NaOH, 10 mM Tris,1% Triton X-100, pH=10) for 40 min. After the lysis stage, slides were rinsed three times with deionized water; then, they were placed in alkaline buffer (0.3 M NaOH, 1 mM EDTA, pH > 13) for 40 min to break DNA into more distinctive parts and to unwind it. Slides were juxtaposed in horizontal electrophoresis power tank containing the same buffer to run electrophoresis for 40 min, at 300 mA and 25 V. Having been neutralized with buffer for 10 min, slides were dried on an arid surface. Finally, slides were stained with ethidium bromide (20 μg/ml, Sigma-Aldrich, USA). To decrease damages to DNA during comet assay, dimmed light and darkness was included.

Observation at 400x magnifications followed the stagnation using a fluorescent microscope, equipped with an excitation filter of 515 to 560 nm and a barrier filter of 590 nm. Subsequently, analysis was permitted using images taken by attached video camera to the microscope, connected to the personal computer, using TriTek Cometscore version 1.5. Finally, to analyze the data statistically, 100 cells were randomly selected (Etebari et al., 2012).

**Statistical analysis**

To perform all statistical analyses, the raw data was processed using IBM - SPSS software 21.0.0 (USA). To express DNA damage, three parameters, namely tail moment, tail length and percent of DNA in tail were used. Then, One-way analysis of variance (ANOVA) followed by Tukey post hoc test was performed. Difference was significant if p≤ 0.05.

## Results

**Genotoxic concentration of irinotecan**

**Figure1:**
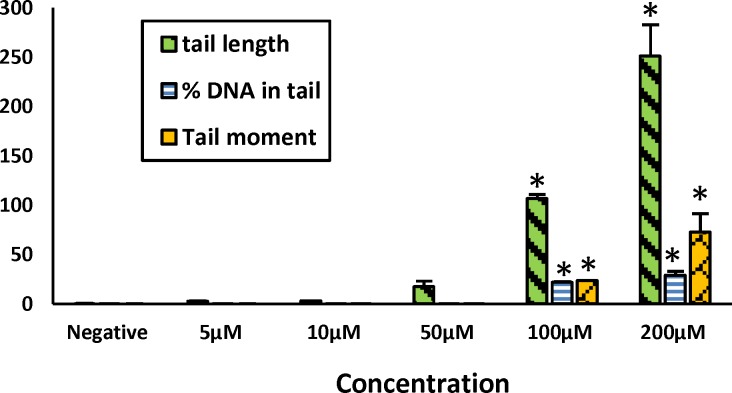
The effect of irinotecan on tail length, %DNA in tail and tail moment of hepG2 cells. Results are presented as mean ± SD from at least three separate experiments, * p< 0.05

In the first phase, comet assay was used to calculate the genotoxic concentration of irinotecan. This test was done at concentrations of 5, 10, 50, and 200 µm and they were compared with control group. Three parameters of tail length, percent of DNA in tail, and tail moment were checked. Results showed significant difference between negative control (HepG2 cells incubated with PBS) and 100 and 200 µM concentrations of irinotecan (p˂0.05). Therefore, 100 µM was selected as the least genotoxic concentration (Figure 1). 

**Safe concentration of heat-killed cells and cell-free supernatants of** ***L. plantarum A7***

To study the protective effects of different concentrations of cell-free supernatants of *L. plantarum A7*, its genotoxic activity at concentrations 500, 100, 10, and 1 µg/ml was investigated primarily. Based on our finding, 1 µg/ml concentration was selected as the safe concentration (p˂0.05).

Similar to the previous stage, genotoxicity of different concentrations of heat-killed cells bacteria was assessed. HepG2 cells were exposed to several concentrations of heat-killed cells of *L. plantarum A7 *(OD620:0.025, 0.05 and 0.1). Results showed significant difference between negative control and the tested concentrations (OD620:0.05 and 0.1). Then, safe concentration (OD620: 0.025) was selected to study the protective effect (Table 1). 

**Table1:** Genotoxic effects of irinotecan, cell-free supernatants and heat- killed cells of *L. plantarum* A7 on HepG2 cells

Treatment		Tail length (mean±SD)	%DNA in tail (mean±SD)	Tail moment (mean±SD)
Negative control (PBS)		1.31 ± 0.45	0.00 ± 0.00	0.00 ± 0.00
Irinotecan concentration (µm)	100	106.54 ± 4.31*	21.92 ± 0.78 *	23.36 ± 0.58 *
Cell free supernatant *of L. plantarum A7* concentration (µg/ml)	500	65.25 ± 3.22 *	25.91 ± 3.91 *	17.69 ± 3.31 *
	100	62.09 ± 3.03 *	17.66 ± 4.73 *	11.46 ± 3.73 *
	10	34.01 ± 1.04 *	11.06 ± 3.00 *	4.92 ± 1.18 *
	1	1.75 ± 0.65	± 0.00	0.00 ± 0.00
	0.5	1.51 ± 0.59	0.03 ± 0.1	0.00 ± 0.00
Heat-kill cell of *L. plantarum A7* OD_620_	0.1	75.18 ± 3.91 *	17.37 ± 2.36 *	14.22 ± 1.07 *
	0.05	67.17 ± 1.18 *	16.06 ± 0.45 *	11.12 ± 0.39 *
	0.025	4.15 ± 0.31	0.00 ± 0.00	0.00 ± 0.00

The experiments were carried out in triplicate. One hundred cells (at least 33 comet scores per experiment) were analyzed per sample. Tail length (pixels), %DNA in tail [DNA tail/ (DNA head + DNA tail)]

* 100 and tail moment (%DNA in tail × length of tail) (pixels) of three independent experiments are represented as mean ± SD. Significant differences between negative control and treatment sample is shown by (*p < 0.05).

**Anti-genotoxicity of cell-free supernatants and heat- kill cells of bacteria**

To study the anti-genotoxicity effect of cell-free supernatants of *L. plantarum A7 *comet assay was performed; HepG2 cells were incubated with cell-free supernatants of *L. plantarum A7 *for 1 hr and exposed to 100 µM irinotecan. Our finding showed that cell-free supernatants of *L. plantarum A7 *at concentrations of 0. 5 and 1 µg/ml significantly reduced irinotecan-induced DNA damage (P˂ 0.05).

Having exposed the cells to 100 µM irinotecan, heat-killed cells of *L. plantarum A7 *were re-incubated with HepG2 cells for 1 hour at OD_620_: 0.025 to test the protective effect of heat-killed cells of bacteria. Results showed that heat-killed cells of *L. plantarum A7 (*OD_620_: 0.025) significantly (p<0.05) reduceed irinotecan-induced DNA damage (Table 2).

**Table 2:** Protective effects of cell-free supernatants and heat- killed cells of bacteria on HepG2 cells treatment with irinotecan

Treatment		Tail length (Mean±SD)	%DNA in tail (Mean±SD)	Tail moment (Mean±SD)
Positive control (Irinotecan) (µm)	100	106.54 ± 4.31	21.92± 0.78	23.36 ± 0.58
Cell free supernatant of * L. plantarum A7* concentration (µg/ml)	1	28.60 ± 0.33*	4.37 ± 1.15 *	1.98 ± 0.68 *
	0.5	35.06 ± 0.73 *	4.44 ± 1.11 *	2.22 ± 0.13 *
Heat-killed cell of * L. plantarum A7* OD_620_	0.025	41.65 ± 4.97 *	11.73 ± 4.38 *	8.51 ± 4.83 *

The experiment was carried out in triplicate. One hundred cells (at least 33 comet scores per experiment) were analyzed per sample. Tail length (pixels), %DNA in tail [DNA tail/ (DNA head + DNA tail)]

* 100 and tail moment (%DNA in tail × length of tail) (pixels) of three independent experiments are represented as mean ± SD. Significant differences between positive control (irinotecan 100 µM ) and treatment sample is shown by (*p< 0.05).

## Discussion

Clinical data have shown associations between irinotecan and histological changes in the liver. Very little is known about the precise mechanisms of irinotecan hepatotoxicity. It seems that accumulation of fat within the hepatocytes following oxidative stress caused by irinotecan results in the development of hepatotoxicity. It is thought that mitochondrial dysfunction causes increased production of ROS through damaged respiratory chain, increased lipid peroxidation and impairment of beta-oxidation. This can trigger release of pro-apoptotic (TNF- α) and pro-fibrotic (TGF-β) cytokines by Kupffer cells leading to cell death, inflammation and fibrosis. It has also been suggested that impairment of mitochondrial topoisomerases and subsequent inhibition of mtDNA replication are potential mechanisms of irinotecan-induced steatohepatitis (Cai et al., 2014).

Several studies have indicated that probiotics can be effective in treating hepatic diseases due to their potential ability to modulate alterations in the gut microbiota, intestinal permeability, and immune and inflammatory responses. Probiotics decrease hepatic steatosis through lowering the hepatic lipid content and low-grade systemic inflammation. The mechanisms of attenuation of hepatic steatosis and liver injury by probiotics are sterol regulatory element-binding protein (SREBP-1) down regulation and peroxisome proliferator-activated receptor-α (PPAR-α) up-regulation (Chávez-Tapia et al., 2015).

The combined form of probiotics composed of *Bifidobacterium*, *Lactobacillus* and *Streptococcus *has a potent antioxidant activity. It can reduce the inflammatory response, expression of PPAR-α and activities of metalloproteinases 2 and 9, and cyclooxygenase that lead to insulin resistance and control fatty acid β-oxidation. These probiotics also reversed high-fat-diet-induced depletion of hepatic natural killer T cells, which resulted in attenuation of TNF-α and IκB kinase inflammatory signaling (Vajro et al., 2011). 

In the present study, cell-free supernatants at concentrations of 0.5 and 1 µg/ml and heat-killed cells of *L. plantarum A7* (OD620: 0.025) significantly reduced irinotecan-induced DNA damage. This attenuation of DNA damage can be attributed to antioxidative and anti-inflammatory properties of the *L. plantarum.*

The results of this study were in line with the results of Burns and Rowland stating that probiotic microorganisms posed protective effects on DNA damages induced by the genotoxicity of fecal water. However, in that study, the given protective effect was revealed to be highly dependent on the probiotics cell concentration as *L. plantarum* cell densities of ≤1.5 × 10^6 ^cfu/ml had little or no inhibitory effect on faecal water genotoxicity (Burns & Rowland 2004). They revealed that the protective effect was also dose-dependent; doses of *L. acidophilus* representing 50 and 10% of the original dose were less effective in reducing MNNG-induced DNA damage (Burns and Rowland 2004). Pool-Zobel et al., reported that *Lactobacillus casei* and *Lactobacillus lactis* decreased the genotoxicity activity of nitrosated beef significantly whereas *Lactobacillus confusus* and *Lactobacillus sake* had no effect (Pool‐Zobel et al., 1993). It was also found that both cellular fractions and produced metabolites by the tested strains could inhibit genotoxic activity.

The anti-genotoxicity of probiotics has been extensively investigated and it was indicated that lactobacilli and other intestinal bacteria can suppress genotoxic damage of dietary carcinogens, *in vitro* (Apás et al., 2014). 

It was mentioned that anti-mutagenic activity of probiotics may be mediated through inhibition of binding the mutagens on the cell surface and peptidoglycans (sugar and protein moieties). In addition, in previous studies, degradation of mutagenesis, detoxification and biotransformation of procarcinogens and carcinogens into less toxic metabolites, lowering intestinal pH by short chain fatty acids (SCFA) production during non-digestible carbohydrate degradation, and activation of the host innate immune system through secretion of anti-inflammatory molecules were all shown to be associated with anti-carcinogenic effects of probiotics (Raman et al., 2013). 

Using animal models for discovering probiotic microorganisms with potential anti-genotoxicity activities is a time-consuming costly effortful process. Comet assay, an *in vitro* methods, is a sensitive, rapid, and simple tool provides a more practical alternative. It has been generally accepted for evaluating DNA damage, repair studies, genotoxicity testing, and human bio-monitoring. The use of irinotecan in conjunction with the comet assay and HepG2 cell lines thus provides a useful and highly relevant *in vitro* model of investigation of *L. plantarum A7* anti-genotoxicity activity (Razavi-Azarkhiavi et al., 2014).
